# Constitutional frequencies of c-Ha-ras alleles in patients with different types of lung cancer.

**DOI:** 10.1038/bjc.1990.33

**Published:** 1990-01

**Authors:** G. R. White, M. Santibáñez-Koref, J. Heighway, N. Thatcher


 © Macmillan Press Ltd., 1990

LETTER TO THE EDITOR

### Constitutional frequencies of c-Ha-*ras* alleles in patients with different types of lung cancer

**Sir** – In a study of constitutional restriction fragment length polymorphisms at the HRAS1 locus in patients with small cell (SCLC) and non-small cell cancer (NSCLC) of the lung and in a control group we reported earlier differences in the distribution of the common alleles between these three groups (Heighway *et al.*, 1986). The main difference was a relative increase of the number of individuals carrying the a4 allele among NSCLC patients (19/66, i.e. 29%) compared with the control group (15/101, i.e. 15%, $P < 0.05$). Patients with SCLC showed a slight decrease in the frequency of this allele (5/66, i.e. 8%, $P < 0.004$ compared with the group of NSCLC patients). We suggested that the allele status may confer a genetic predisposition to a particular type of lung cancer.

In order to test the reproducibility of those results we analysed a new set of 238 patients. The only difference between both studies is that in the present one the DNA was exclusively extracted from peripheral blood, whereas in the first study in some cases only tumour material was used. The present results do not show, at a probability level of 0.05, any significant difference between the three groups in the allele frequencies (Table I) or in the proportions of individuals carrying the a4 allele (Table II).

Our new data fail to support the hypothesis that certain c-Ha-*ras* alleles are involved in a genetic predisposition to certain types of lung cancer.

**Table II** Individuals carrying the a4 allele

| | | |
|---|---|---|
| Controls | 15/101 | (15%) |
| SCLC patients | 18/137 | (13%) |
| NSCLC patients | 13/101 | (13%) |

Yours, etc.

G.R.M. White, M. Santibáñez-Koref, J. Heighway,
Paterson Institute for Cancer Research,
N. Thatcher
CRC Department of Medical Oncology,
Christie Hospital and Holt Radium Institute,
Wilmslow Road,
Manchester M20 9BX, UK.

ACKNOWLEDGEMENT

THIS WORK WAS SUPPORTED BY THE CANCER RESEARCH CAMPAIGN.

**Table I** Allele distribution of c-Ha-*ras* in 238 patients with small cell (SCLC) and non-small cell cancer (NSCLC) of the lung

| | *Allele type* | | | | | |
|---|---|---|---|---|---|---|
| | *a1* | *a2* | *a3* | *a4* | *Rare* | *Total* |
| SCLC | 172 (63%) | 33 (12%) | 34 (12%) | 18 (7%) | 17 (6%) | 274 |
| NSCLC | 124 (61%) | 26 (13%) | 30 (15%) | 14 (7%) | 7 (3%) | 202 |
| Controls | 120 (60%) | 32 (16%) | 26 (13%) | 15 (7%) | 9 (4%) | 202 |

The data for the control group were taken from Heighway *et al.* (1986). The alleles were determined using Pvu II digests of genomic DNA and the probe pT24-C3 (Reddy *et al.* 1982).

### References

HEIGHWAY, J., THATCHER, N., CERNY, T. & HASELTON, P.S. (1986). Genetic predisposition to human lung cancer. *Br. J. Cancer*, **53**, 453.

REDDY, E.P., REYNOLDS, R.K., SANTOS, E. & BARBACID, N. (1982). A point mutation is responsible for the acquisition of transforming properties by the T24 human bladder carcinoma oncogene. *Nature*, **300**, 149.


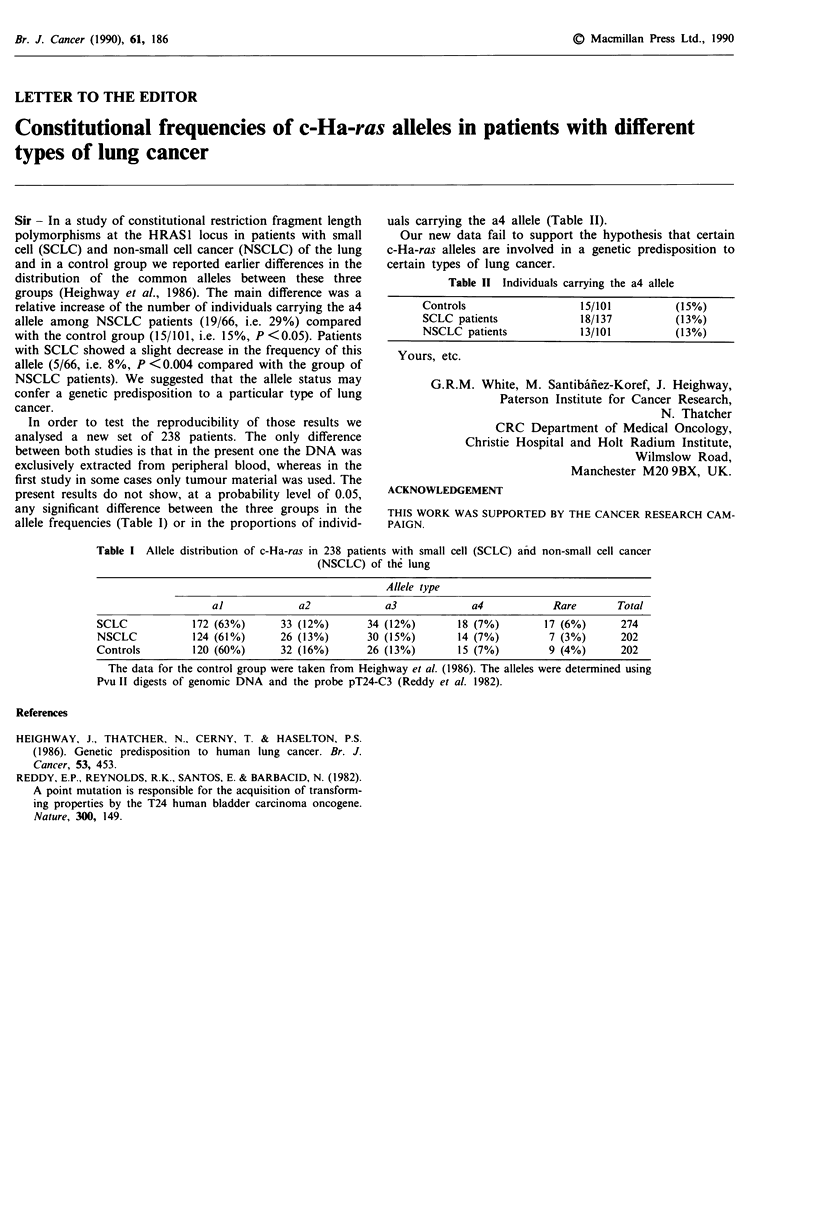


## References

[OCR_00076] Heighway J., Thatcher N., Cerny T., Hasleton P. S. (1986). Genetic predisposition to human lung cancer.. Br J Cancer.

[OCR_00081] Reddy E. P., Reynolds R. K., Santos E., Barbacid M. (1982). A point mutation is responsible for the acquisition of transforming properties by the T24 human bladder carcinoma oncogene.. Nature.

